# Aerosol Synthesis of N and N-S Doped and Crumpled Graphene Nanostructures

**DOI:** 10.3390/nano8060406

**Published:** 2018-06-06

**Authors:** Francesco Carraro, Mattia Cattelan, Marco Favaro, Laura Calvillo

**Affiliations:** Dipartimento di Scienze Chimiche, Università degli Studi di Padova, 35122 Padova, Italy; mattia.cattelan@bristol.ac.uk (M.C.); marco.favaro@helmholtz-berlin.de (M.F.); laura.calvillolamana@unipd.it (L.C.)

**Keywords:** graphene, chemically modified graphene, crumpled graphene, nitrogen doping, sulfur doping, dual doping, aerosol synthesis

## Abstract

Chemically modified graphene–based materials (CMG) are currently attracting a vast interest in their application in different fields. In particular, heteroatom-doped graphenes have revealed great potentialities in the field of electrocatalysis as substitutes of fuel cell noble metal–based catalysts. In this work, we investigate an innovative process for doping graphene nanostructures. We optimize a novel synthetic route based on aerosol preparation, which allows the simultaneous doping, crumpling, and reduction of graphene oxide (GO). Starting from aqueous solutions containing GO and the dopant precursors, we synthesize N- and N,S-dual-doped 3D graphene nanostructures (N-cGO and N,S-cGO). In the aerosol process, every aerosol droplet can be considered as a microreactor where dopant precursors undergo thermal decomposition and react with the GO flakes. Simultaneously, thanks to the relatively high temperature, GO undergoes crumpling and partial reduction. Using a combination of spectroscopic and microscopic characterization techniques, we investigate the morphology of the obtained materials and the chemical nature of the dopants within the crumpled graphene sheets. This study highlights the versatility of the aerosol process for the design of new CMG materials with tailored electrocatalytic properties.

## 1. Introduction

During the last few years, graphene, a monolayer of carbon sp^2^ atoms arranged in a honey-comb network, and chemically modified graphene (CMG) systems have been studied in the context of several applications, due to their excellent chemical, electrical, mechanical and thermal properties [1,2,3,4]. In particular, the introduction of heteroatoms in the graphene structure can impart new functionalities and new chemical properties to the graphene-based materials.

One of the fields of great interest for CMG is energetics and, in particular, the research for valid substitutes of platinum for the oxygen reduction reaction (ORR) occurring at the cathodic side of fuel cells [5]. Both theoretical and experimental studies have proven that heteroatom-doped carbon materials with a graphitic structure show pronounced catalytic activity for ORR, opening a way to the production of metal-free catalysts [6]. Dual–doped CMG materials are particularly fascinating and represent the forefront of metal-free electrocatalyst research [7]. Among the possible heteroatoms that can be introduced into the graphene lattice, sulfur (S) and nitrogen (N) have provided the most promising results in this field [8,9,10]. To perform the graphene doping with heteroatoms, several methods are present in the literature including: chemical vapor deposition (CVD) [11,12], annealing in gas precursor (i.e., NH_3_, H_2_S) [13], hydrothermal [14], electrochemical [15] or microwave [16] methods.

The most consolidated strategies to include heteroatoms in graphene or to create new nanohybrid materials, which are composite systems constituted by graphene and other nano-objects (e.g., nanoparticles), usually start from graphite oxide [17,18,19]. Graphite oxide is a compound of carbon, oxygen and hydrogen that, when it is dispersed in a solution, yields single graphenic sheets called graphene oxide (GO) in analogy to graphene. GO is a quite promising raw material due to its easy handling and manipulation (i.e., it is water soluble, producible in high yield, easy to assemble into different morphology) [20,21,22]. However, one of the principal issues concerning the use of these 2D nanosheets is their natural tendency to aggregate (restacking) due to strong inter-sheet adhesion (Van der Waals attraction). As a consequence of restacking, there is a reduction of the accessible surface area that compromises the advantage of the 2D structure [23]. To avoid restacking, 3D graphene structures can be obtained, for instance, by thermal treatments of GO coated polymer foams [24], soft-template-casted melamine foams [25] or freeze-dried GO [26]. Alternatively, carbon precursors impregnated mesoporous silica can be thermally treated to obtain the carbon replica of the porous structure [27]. In this manner, by adding a heteroatoms precursor it is possible to obtain S-N doped 3D and porous graphene structures as well. In this context, particularly attractive is the so-called “crumpled” form of GO (i.e., micrometric GO sheets collapsed to form paper-like nanospheres [28]), since the wrinkling of GO sheets allows obtaining special active sites (i.e., strain-induced chemical activity [29]) and high surface area [30] while maintaining good electrical conductivity [31].

Aerosol processing is one of the most promising techniques to obtain crumpled graphene oxide (cGO) [32,33,34]. This method is simple, environmentally-friendly if it starts from aqueous solution, characterized by short processing time and can be applied in continuous (and scalable) production. Due to its high hydrophilicity, GO forms stable dispersions in water, thereby emerging as a suitable candidate for green aerosol processing into CMG–based or hybrid materials. [35,36].

In this paper, we have exploited aerosol processing as an alternative route for the doping of graphene based nanostructures. In a recent work, we have reported for the first time the study of this kind of one-pot process which combines crumpling, reduction and N doping of GO for the synthesis of CMG–based hybrid materials (N-cGO/MoS_2_) [37]. In the present study, we have focused our attention on the one-step crumpling/doping process and the possibility to obtain dual N,S-doped cGO. We carefully investigated the aerosol synthesis of N- and N,S-doped cGO systems(N-cGO and NS-cGO, respectively), using a combination of microscopic and spectroscopic characterization techniques. Our findings show that during the flight of aerosol droplets through the hot furnace, the dopant precursors undergo thermal decomposition into highly reactive gaseous species (e.g., NH_3_ and H_2_S) [38,39] that can react with graphene thereby leading to chemical doping. At the same time, the relatively high temperature induces the crumpling and the partial reduction of the GO sheets. The result is the formation of doped cGO. The ability to combine chemical doping with highly curved graphene sheets represents an interesting solution to obtain novel materials characterized by a high surface area and improved chemical and catalytic activity.

## 2. Materials and Methods

### 2.1. Synthetic Procedures

We investigated the synthesis conditions for the preparation of crumpled GO (cGO), nitrogen-doped cGO (N-cGO) and nitrogen–sulfur-doped cGO (NS-cGO) by the aerosol process. GO starting material was synthesized following a modified Hummer method [40] (briefly reported in the Supporting Information). All synthesis protocols are based on the formation of an aerosol operated by an ultrasonic nebulizer (Sonaer 241PG 2.4 MHz, West Babylon, NY, USA; particle size about 1.7 ± 1 μm) starting from an aqueous precursor solution. The precursor solutions for the different materials are reported in Table 1. Particles generated by the ultrasonic nebulizer are forced to pass through a furnace, set at 800 °C, using N_2_ or N_2_/H_2_ (9:1, v/v) as the carrier gas. Hereafter, depending on the reaction atmosphere, the samples will be labeled as N_2_ or N_2_ + H_2_, respectively. The experimental set-up is schematized in Scheme S1. Crumpling process takes place during the whole time-of-flight of the aerosol droplets, but mainly during the passage through the hot furnace where water evaporation is favored. Si (100) wafers, gold coated silica filters (EPM2000, Whatman, Maidstone, UK) and Toray paper (TGP-H-60, carbon-fiber-based filters, Alfa Aesar, Haverhill, MA, USA) were used as substrates for collecting the particles. Typical collection time was 30 min.

### 2.2. Structural Characterization Tools

X-ray Photoelectron Spectroscopy (XPS, VG Scienta, Taunusstein, Germany) spectra were acquired using a non-monochromatic Al K_α_ X-rays source (hν = 1486.6 eV). Previously to the measurements, the samples were subject to 30 min of annealing at 100 °C in ultra-high vacuum (UHV), mainly to desorb water and loose contaminants. The calibration of the binding energy (BE) scale was determined using the Au 4*f*_7/2_ core level spectrum (BE = 84.0 eV). The XPS peaks were separated into chemically shifted components (after Shirley background removal) using symmetrical pseudo Voigt functions and non-linear least squares routines for the χ^2^ minimization. The nano- and micro-scale morphology was studied by scanning electron microscopy (SEM, Zeiss Supra VP35, Oberkochen, Germany). SEM images were acquired using a field emission source equipped with a GEMINI column, micrographs were obtained with an acceleration voltage of 5 or 10 kV using in-lens high-resolution detection. Characterization by Raman spectroscopy was performed using a ThermoFisher (Waltham, MA, USA) DXR Raman microscope. The spectra were recorded using a laser with an excitation wavelength of 532 nm (1 mW), focused on the sample with a 50× objective (Olympus, Shinjuku, Japan). The Raman spectra were deconvoluted using pseudo Voigt and non-linear least squares routines for the χ^2^ minimization in the range 1000–2000 cm^−1^.

## 3. Results and Discussion

In this work, we investigated a one-pot aerosol synthesis for the simultaneous reduction, crumpling and chemical doping of GO. Notably, all materials were prepared using the same synthesis protocol (e.g., forming an aerosol transported by an N_2_ or H_2_/N_2_ carrier gas through a furnace kept at 800 °C). The doping was achieved by adding the heteroatom precursors to the solution that undergoes aerosolization (see Scheme S1), i.e., NH_4_OH [41] to induce N doping and l-cysteine or thiourea [42] to induce N,S-dual doping. Indeed, with this method, each aerosol microdroplet can be treated as a microreactor, similar to microemulsions [43], supercritical hydrothermal [44] or templated chemical syntheses [45]. The doped materials were compared to undoped cGO synthesized at the same temperature, to verify the eventual effect of the doping on the morphology and degree of reduction of the crumpled particles. The structural and chemical properties of these systems were thoroughly investigated by spectroscopic and microscopic techniques to understand the influence of reaction atmosphere and precursors on the final materials.

**Figure 1:**
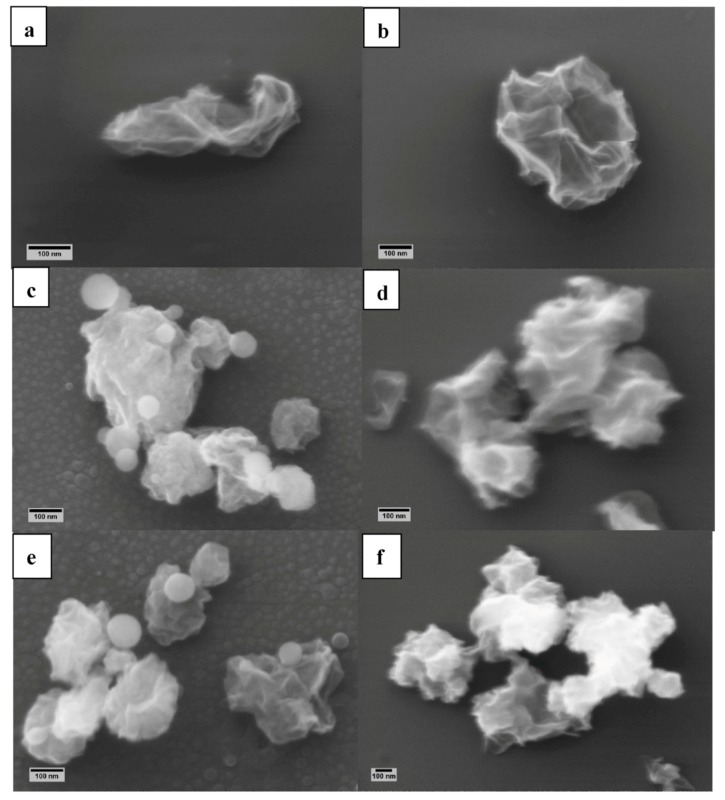
SEM micrographs of (**a**) N-cGO (N_2_); (**b**) N-cGO (N_2_ + H_2_) (precursor: ammonium hydroxide), (**c**) NS-cGO (N_2_) and (**d**) NS-cGO (N_2_ + H_2_) (precursor: l-cysteine), (**e**) NS-cGO (N_2_) and (**f**) NS-cGO (N_2_ + H_2_) (precursor: thiuorea) collected on Si(100) wafers.

SEM investigations were performed to study the microscopic structure of the obtained materials. Figure 1 reports the micrographs of N-cGO obtained from ammonium hydroxide and NS-cGO obtained from l-cysteine and thiourea under N_2_ (Figure 1a,c,e) and N_2_ + H_2_ gas flow (Figure 1b,d,f) collected on Si (100) substrates. The collection on Si substrates is very useful for single particle morphologic studies, which are crucial for fundamental studies addressing new and complicated structures like the crumpled materials, and more in general for the individuation of precise links between process parameters and structure of the prepared materials. Nevertheless, to have a more efficient, and possibly complete collection of generated particles, the whole flow can be intercepted with a filter (i.e., Figures S2 and S3).

**Figure 2:**
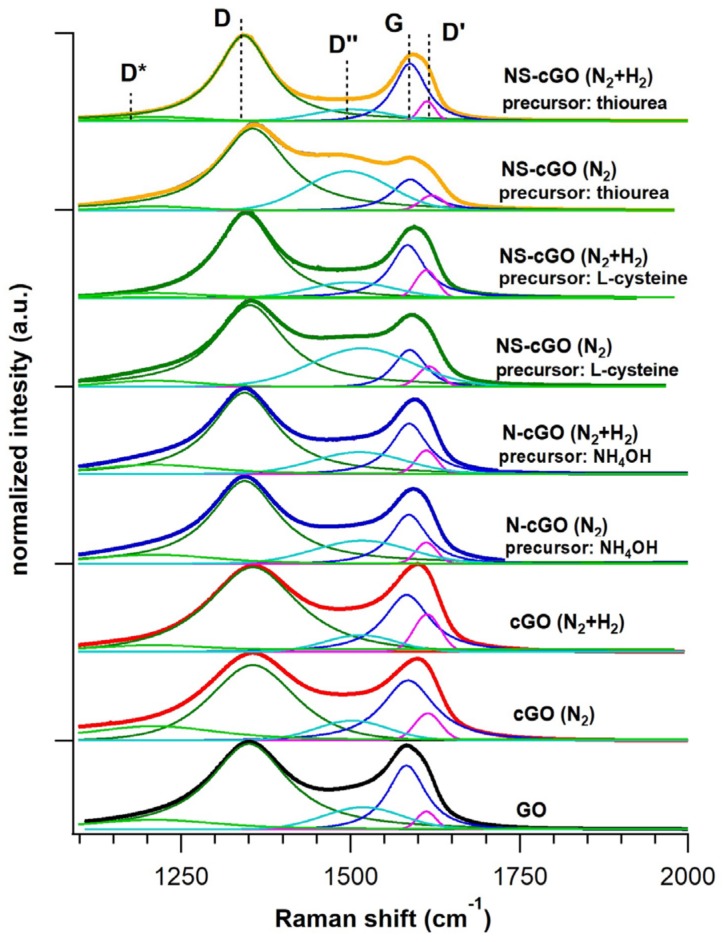
Raman spectra (1100–2000 cm^−1^), as well as their deconvolution, of the starting GO, the undoped cGO and the N-(S-) doped samples.

By analyzing the SEM micrographs, it results that the doped cGO particles size ranges from 100 to 400 nm in all the samples and that all the particles are morphologically similar and comparable to undoped cGO particles synthesized in inert atmosphere at the same temperature (Figure S2b). However, in the case of NS-cGO (N_2_) samples, a second population of small spherical particles (average diameter < 100 nm) is also present (Figure 1c,e). These particles may be associated with amorphous carbon containing N and S species due to ineffective/partial decomposition of the doping precursors within the microreactors (i.e., aerosol droplets). In fact, from the Raman spectra (discussed in detail below, Figure 2), it is not possible to identify the typical fingerprint of l-cysteine or thiuorea, confirming that these spherical particles were not related to agglomerates of the undecomposed starting precursor source molecules. However, it is possible to notice an increase of the intensity in the region 1450–1550 cm^−1^ that may be ascribable to the presence of amorphous carbon [46].

To remove these unwanted species, we changed the reaction atmosphere from inert to reductive by using a mixture of molecular nitrogen and hydrogen as a gas carrier (9/1 volume ratio). The other process parameters were not changed (i.e., composition of the starting solutions, furnace temperature). The SEM micrographs of the various samples synthesized in reductive atmosphere (Figure 1b,d,f) show that the morphology of the crumpled particles is comparable to doped and undoped cGO synthesized in inert environment. Notably, in the case of NS-cGO (N_2_ + H_2_) samples, SEM micrographs did not show the small spherical particles observed in the case of NS-cGO (N_2_) samples (Figure 1d,f). Moreover, to verify that the crumpling process was not affected by the presence of hydrogen, we carried out a synthesis in the new gas mixture without doping precursors. The SEM micrographs (Figure S2) did not show any variation in the crumpled structure and Raman spectra were comparable with the results obtained in inert atmosphere (Figure 2). Moreover, from the analysis of XPS data, we verified that C 1s spectrum of cGO (N_2_ + H_2_) is almost identical to the C 1s spectrum of cGO (N_2_) (Figure S1). This confirmed that the presence of hydrogen in the reaction atmosphere did not affect the crumpling process of graphene oxide sheets.

XPS represents the best methodological approach to provide a specific chemical assignment of the defects type (e.g., to discriminate between oxidized or reduced components) and a possible interaction between them (N–S, N–O, S–O), because of the significant chemical shifts that can be observed in the respective core level photoemission lines [47]. Moreover, the chemical states of the dopants have a key role in determining the charge and spin density in the material, which are considered some of the key parameters controlling the catalytic activity of doped graphene materials.

**Figure 3:**
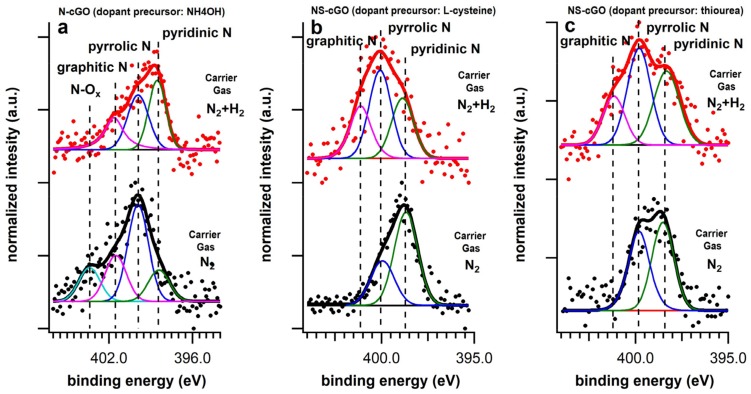
N 1s photoemission lines, as well as the single chemically shifted components, of the sample synthesized in inert (black lines) and reductive atmosphere (red lines) using (**a**) ammonium hydroxide (N-cGO); (**b**) l-cysteine (NS-cGO) and (**c**) thiuorea (NS-cGO) as doping precursor.

**Figure 4:**
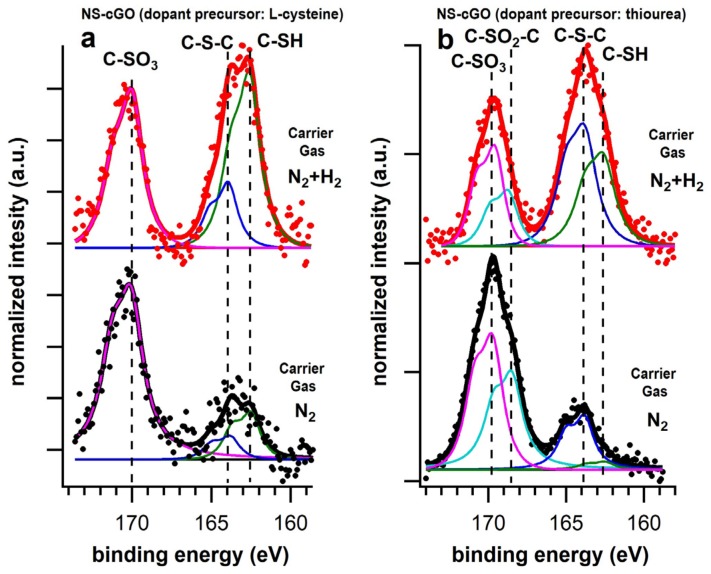
S 2p photoemission lines, as well as the single chemically shifted components, of the sample synthesized in inert (black lines) and reductive atmosphere (red lines) using (**a**) l-cysteine (NS-cGO) and (**b**) thiuorea (NS-cGO) as doping precursor.

As mentioned above, the syntheses were carried out under inert and reductive atmospheres, using N_2_ or N_2_ + H_2_ as carrier gases. Table 2 reports the C/dopants stoichiometry for all the prepared systems calculated from the XPS data. The surface atomic percentage of nitrogen (N%) in the N-cGO (N_2_) sample is similar in terms of dopant concentration to those already reported in the literature for similar materials. In this case, the N 1s photoemission line was deconvoluted into four single chemically shifted components (Figure 3a). The most intense peak, centered at 400 eV, is associated with pyrrolic defects [48,49]. The peaks centered at 398.5 eV and 401.5 eV are related to pyridinic and N substitutional defects, respectively. NO_x_ defects (403.2 eV) represent the less intense component (15% of the total integrated area). Conversely, in the case of both NS-cGO (N_2_) samples (Figure 3b,c), the N% and S% were higher than expected and the N 1s photoemission lines were fitted with only two components at 400 eV (pyrrolic defects) and 398.5 eV (pyridinic defects). In both cases, the pyridinic defects were the majority components. However, neither N substitutional defects nor oxidized N species were detected in these cases (Table S1). Considering the analysis of S 2*p* photoemission lines, we verified that the presence of sulfur was mainly related to oxidized S species (80% of the total integrated area in both cases, Figure 4 and Table S1). In fact, in the multipeak analysis of the S 2*p* photoemission lines (Figure 4) the main components are localized at 169.8 eV (both samples) and 168.5 eV (thiourea sample), corresponding to –SO_3_ and –C–SO_2_–C groups, respectively. Thiophenic-like units (–C–S–C–, 164 eV) and –SH groups (162.5 eV) represent only the 20% of the total integrated area and were detected in both samples.

**Figure 5:**
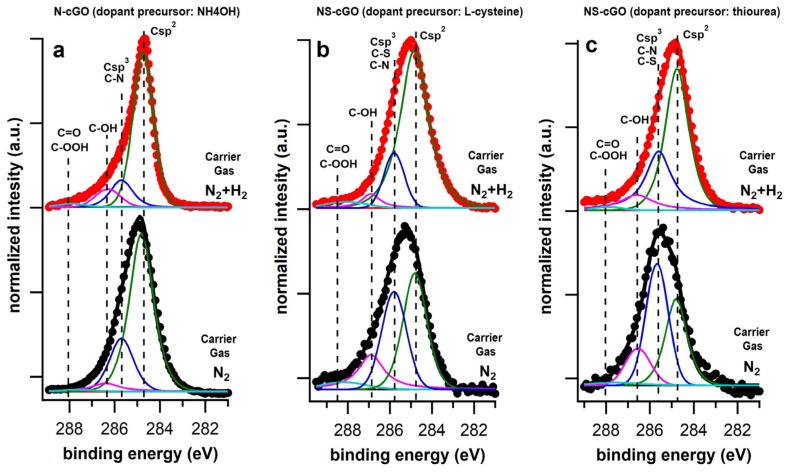
C 1s photoemission lines, as well as the single chemically shifted components, of the sample synthesized in inert (black lines) and reductive atmosphere (red lines) using (**a**) ammonium hydroxide (N-cGO); (**b**) l-cysteine (NS-cGO) and (**c**) thiuorea (NS-cGO) as doping precursor.

The C 1s photoemission lines (Figure 5) were separated into four chemically shifted components for all samples, according to the standard procedure reported in the literature [50]. In the case of N-cGO (N_2_) sample (Figure 5a), the most intense peak, centered at 284.7 eV, is associated with sp^2^ hybridized carbon atoms. The peak at 285.7 eV can be related either to sp^3^ hybridized carbon component, which is connected to the crumpling of the sheets and the consequent creation of defects, or to C−N bonds. Finally, the features centered at about 286.6 and 288 eV are associated with tertiary alcohols and epoxy/carbonyl groups, respectively. Because of the GO reduction during the time-of-flight of the microdroplets inside the hot furnace, these three components are strongly reduced with respect to pristine GO (see the C 1s spectrum reported in Figure S1). In the case of NS-cGO (N_2_) samples (Figure 5b,c), the multipeak analysis of C 1s photoemission lines showed that the component related to both sp^3^ hybridized carbon and C–N/C–S bonds (285.7 eV) is very intense. Moreover, the component related to tertiary alcohols is also more intense than in the case of N-cGO (N_2_) sample.

Taking into account the samples morphology observed by SEM, we can ascribe the high percentage of N and S and the high amount of sp^3^ hybridized carbon in the NS-cGO (N_2_) samples to the presence of the carbonaceous spherical particles originated by an ineffective decomposition of l-cysteine and thiourea to highly reactive species (i.e., NH_3_, SH_2_) under our experimental conditions.

Looking at the C/dopants stoichiometry calculated from XPS data and reported in Table 2 for the samples synthesized under reductive atmosphere, the absence of the small spherical particles results in a lower N% and S% in both NS-cGO (N_2_ + H_2_) samples. In fact, by introducing hydrogen in the reaction atmosphere, the dopant concentrations in the N-cGO samples and in the NS-cGO (N_2_ + H_2_) samples are in line with those already reported in the literature for similar materials [14]. Therefore, we can ascribe the signals of N and S in the NS-cGO (N_2_ + H_2_) samples as dopants within the crumpled graphene sheets.

In these samples, the N 1s photoemission line (Figure 3b,c) can be fitted with three components: 400 eV (pyrrolic defects), 398.5 eV (pyridinic defects) and N substitutional defects (401.5 eV). In both cases, the pyrrolic defects were the majority components. However, the component related to pyrydinic defects is slightly more intense in the case of the sample synthesized using thiourea. Oxidized N species were not detected in the experiments using H_2_. From the multipeak analysis of S 2*p* photoemission lines, we verified that oxidized S species (169.8 and 168.5 eV) were still present on the samples, but their amount was considerably lower than in the samples synthesized under inert atmosphere (between 40% and 35% of the total integrated area, Figure 4 and Table S1). Thiophenic-like units (–C–S–C–, 164 eV) and –SH groups (162.5 eV) represent the majority species of the total integrated area in these cases.

The nature of the N-S precursor influenced the ratio between the S and N species on the final material. The effect on the N species was already discussed above; regarding S species, the main S component is related to –SH groups when l-cysteine is used as the precursor. Conversely, when thiourea is used as the precursor, the component related to thiophenic-like units is slightly more intense than the one related to –SH groups. Considering the multipeak analysis of the C 1s photoemission lines (Figure 5b,c), the absence of the spherical particles leads to an important decrease of the component related to both sp^3^ hybridized carbon and C-N/C-S bonds (285.7 eV). In fact, in the C 1s spectra of the NS-cGO (N_2_ + H_2_) samples the component associated with sp^2^ hybridized carbon is the most intense one, as expected for this kind of materials. Finally, considering the NS-cGO (N_2_ + H_2_) samples, it is worth mentioning that the S/N ratio in the dual doped samples is very close to the S/N ratio in the precursors (1:1 for l-cysteine and 1:2 for thiourea, see Table 2), indicating that it can be easily tuned by just selecting a suitable precursor.

The N 1s photoemission line for the N-cGO (N_2_ + H_2_) sample, together with its deconvolution into single chemically shifted components, is reported in Figure 3a. Unlike the N-cGO (N_2_) sample, only three different components can be identified: pyridinic (the most intense), pyrrolic and N substitutional defects. In this case, the introduction of hydrogen in the reaction atmosphere avoid the formation of oxidized N species (the component at around 403 eV was not detected). Considering the C 1s photoemission line (Figure 5a), the main peak is associated with sp^2^ hybridized carbon (284.7 eV) and, compared to the N-cGO (N_2_) sample, we can notice a slight reduction of the intensity of the peak centered at 285.7 eV. This might be associated with the slight lower N% compared to the sample synthesized in inert atmosphere (2% vs. 2.5%).

In addition to XPS and SEM, we characterized all the samples by Raman spectroscopy, which is a powerful technique for the investigation of graphene-based materials due to their well-known spectral features (Figure 2). GO shows characteristic fingerprint that depends on its reduction degree and its defectivity. All the Raman spectra present the characteristic D (~1350 cm^−1^), G (~1600 cm^−1^), 2D (~2700 cm^−1^) and D + D′ (~2900 cm^−1^) bands of graphene [51]. To carefully investigate the contribution of amorphous carbonaceous particles, the position and FWHM of D and G band and their intensity ratio, the Raman spectra were deconvoluted following the procedure reported in [46] (see Figure 2 and Table S2). As mentioned above, the peak intensity in the region 1450–1550 cm^−1^ (D″ band) is higher in NS-cGO (N_2_) samples than in N-cGO (N_2_) and cGO (N_2_) ones and this is associated with the presence of amorphous or highly defective carbon [39]. In the NS-cGO samples synthesized in reductive atmosphere, the contribution of the D band is heavily reduced and this can be associated with the suppression of the amorphous carbonaceous particles formation. The intensity ratio between D and G (I_D_/I_G_), is a useful parameter to evaluate the reduction degree of. The position and FWHM of D and G bands and the I_D_/I_G_ ratio are influenced by several factors including: doping, strain, reduction or defectivity [52]. The study of all these effects is out of the scope of this paper, however we can highlight some trends in the investigated samples. Comparing the as-prepared GO and cGO, we can see that there is an increase of the I_D_/I_G_ ratio (from 1 to 1.2, see Table S2). Even in the case of doped cGO, I_D_/I_G_ is higher (about 1.1) compared to GO. Indeed, the reduction of GO increased the sp^2^ patches into the layer, inducing a decrease of the D band and consequently a reduction of the I_D_/I_G_ ratio. On the contrary, the increase of D/G band intensity ratio could be attributed to defects introduced by the crumpling process or by the presence of N or S defects that locally break the carbon sp^2^ lattice symmetry. Compared to the Raman spectrum of the as-prepared GO, the G band frequency seems to be shifted to higher Raman shift. However, from the analysis of the deconvoluted spectra, G band Raman shift is not varying significantly (ranging from 1580 to 1585 cm^−1^). The apparent shift is associated with the increase of the intensity of D′ band (1610 cm^−1^), related to disorder-induced phonon modes and the increase of the D band [46].

## 4. Conclusions

In the present work, we have investigated an innovative process for the synthesis of doped and crumpled graphene nanostructures: aerosol synthesis. Starting from an aerosol of aqueous solutions containing GO and the dopants precursors (NH_4_OH, l-cysteine or thiourea) we were able to synthesize N and N-S doped crumpled graphene nanostructures. The aerosol microdroplets can be considered as microreactors. During the time-of-flight inside the hot furnace, heteroatom-containing precursors undergo thermal decomposition and chemical reactions take place between the activated dopant molecules and carbon atoms, thereby leading to the formation of doped material. Notably, the doping occurs simultaneously to the crumpling and reduction processes of GO. Combining the spectroscopic and microscopic characterization technique, we have compared the chemical nature of the doping using inert or reductive atmosphere. We have verified that the use of molecular hydrogen as second carrier gas decreases the presence of oxidized defects in favor of the reduced dopant species. Moreover, hydrogen favors the decomposition of l-cysteine and thiourea in highly reactive intermediates (i.e., NH_3_, H_2_S) useful for the doping of graphene and suppressing the formation of undesired carbonaceous particles.

These materials are attractive for the emerging field of energetics, in particular because of their high activity and selectivity toward the ORR [13,32,53,54]. Furthermore, thanks to their unique chemical and morphological properties these materials are attractive for their potential application as supercapacitors [55,56]. In this context, our study highlights the versatility of the aerosol process for the tailoring of the doping chemical nature (i.e., suppression of inactive oxidized species by changing reaction atmosphere) and the ratio of different heteroatoms in the case of dual doped materials. We think that our synthetic approach can be very useful for the design of new CMG-based electrocatalysts.

## Figures and Tables

**Table 1:** Precursor solutions used for the synthesis of the (doped-)cGO-based materials.

Sample	GO	Doping Precursor	Solvent
cGO	0.5 mg/mL	---	H_2_O
N-cGO	0.5 mg/mL	0.5 M NH_4_OH (Sigma-Aldrich)	H_2_O
NS-cGO	0.5 mg/mL	0.5 M l-cysteine (Sigma-Aldrich)	H_2_O
NS-cGO	0.5 mg/mL	0.5 M thiourea (Sigma-Aldrich)	H_2_O

**Table 2:** Surface atomic percentage (calculated with the respect of C) of N and S in the investigated samples.

Carrier Gas	Precursors				
	NH_4_OH N-cGO	l-cysteine NS-cGO		Thiourea NS-cGO	
	N%	N%	S%	N%	S%
N_2_	2.5	7	12	9	20
N_2_/H_2_ (9/1, v/v)	2	2	2.5	3	1.5

## Supplementary Materials

The following are available online at http://www.mdpi.com/2079-4991/8/6/406/s1. Synthesis of GO. Scheme S1: schematic view of the experimental set-up; Table S1: Resume of the ratios between N and S oxidized species and the total integrated area of N 1s and S 2*p* photoemission lines of the investigated samples; Table S2: D and G bands parameters obtained from the deconvolution of the Raman spectra of GO and of cGO, N-cGO, NS-cGO synthesized in inert and reductive atmosphere; Figure S1: C 1s photoemission lines, as well as the separations into single chemically shifted components, of GO and cGO (N_2_) and (N_2_ + H_2_); Figure S2: SEM micrographs of (a) N-cGO (N_2_ + H_2_) and (b) cGO (N_2_) collected on Toray paper filters; Figure S3: SEM micrographs of N-cGO (N_2_ + H_2_) collected on Toray paper filter.
