# Supplementary material for: Aerosol Synthesis of N and N-S Doped and Crumpled Graphene Nanostructures
Source: Nanomaterials (Basel). 2018 Jun 6;8(6):406. doi: 10.3390/nano8060406 (PMC6027206; doi:10.3390/nano8060406)
Supplement: Supplementary file 1 [file nanomaterials-08-00406-s001.pdf]

Supporting information for

Aerosol Synthesis of N and N-S Doped and Crumpled Graphene Nanostructures

Francesco Carraro [1,*], Mattia Cattelan [1,2], Marco Favaro [1,3] and Laura Calvillo [1]

[1] Università degli Studi di Padova, Dipartimento di Scienze Chimiche, Padova, 35122, Italy;
[2] Present Address: School of Chemistry, University of Bristol, Cantocks Close, Bristol BS8 1TS, UK;
[3] Present Address: Helmholtz-Zentrum Berlin für Materialien und Energie GmbH, Hahn-Meitner-Platz 1, D-14109 Berlin, Germany;
* Correspondence: francesco.carraro.7@phd.unipd.it; Tel.: + 39 049 8275167

Synthesis of GO

The preparation of GO was performed using the modified Hummer's oxidation reaction [1]. Two grams of ultra-pure graphite micrometric powder (d < 150 μm, Sigma-Aldrich, Saint Louis, Missouri, USA) were slowly added to 50 mL of a 9:1 (v/v) H_2SO_4/H_3PO_4 (96% and 65% respectively, Sigma-Aldrich) mixture placed in an ice–water bath. Then, the mixture was transferred to an ice–water–acetone bath and 6 g of $KMnO_4$ (Sigma-Aldrich) were gradually added under vigorous stirring. After 4 h stirring at room temperature, the mixture was heated at 35 °C for 2 h in an ultrasonic bath. After that, the slow addition of 100 mL of deionized water caused an increase in temperature to about 98 °C. The mixture was maintained at this temperature for 15–20 min. The reaction was terminated by adding 280 mL of DI water at 60 °C and, subsequently, 2 mL of 30% H_2O_2 solution (Fluka, Waltham, Massachusetts, USA). After 30 min of stirring and 30 min of sonication, a yellow product was collected after several cycles of centrifugation–water washing to obtain a dispersion of GO in water with a pH of 5.5. This weakly acidic environment is suitable to achieve an optimum stability time of the GO dispersions. The GO dispersion were furtherly purified by means of dialysis (5 days, Spectrapor dialysis membranes MWCO 1000, Spectrum Inc., Rancho Dominguez, California, USA). Finally, the dialyzed GO dispersion were freeze dried in order to obtain GO powder.

Scheme S1. Schematic view of the experimental set-up.

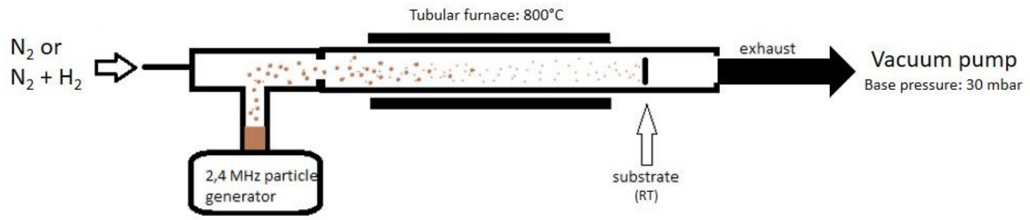

Table S1: Resume of the ratios between N and S oxidized species and the total integrated area of N 1*s* and S 2*p* photoemission lines of the investigated samples.

Carrier Gas	Precursors				
	NH_4OH	L-cysteine		Thiourea	
	NO_x/N_{tot} %	NO_x/N_{tot} %	SO_x/S_{tot} %	NO_x/N_{tot} %	SO_x/S_{tot} %
N_2	15%	0%	80%	0%	80%
N_2/H_2 (9/1, v/v)	0%	0%	40%	0%	35%

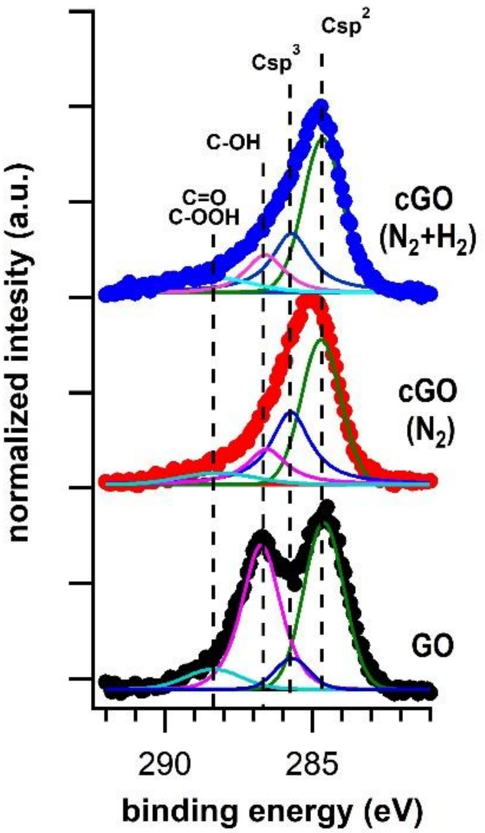

Figure S1. C 1*s* photoemission lines, as well as the single chemically shifted components, of graphene oxide (GO) and of crumpled graphene oxide (cGO) synthesized at 800 °C in N_2 and N_2+H_2 atmosphere.

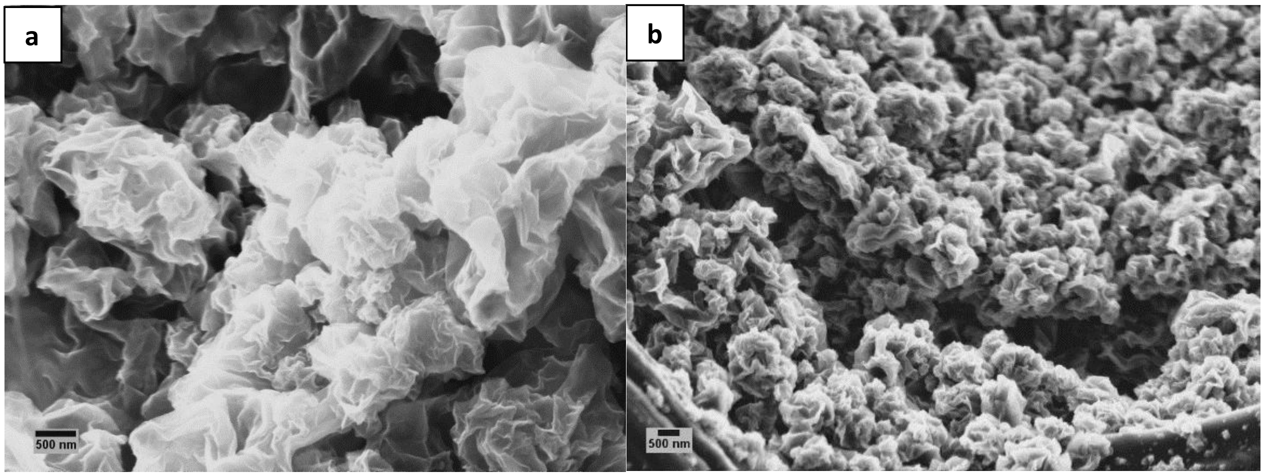

Figure S2. SEM micrographs of (**a**) N-cGO(N_2+H_2) and (**b**) cGO(N_2) collected on Toray paper filters.

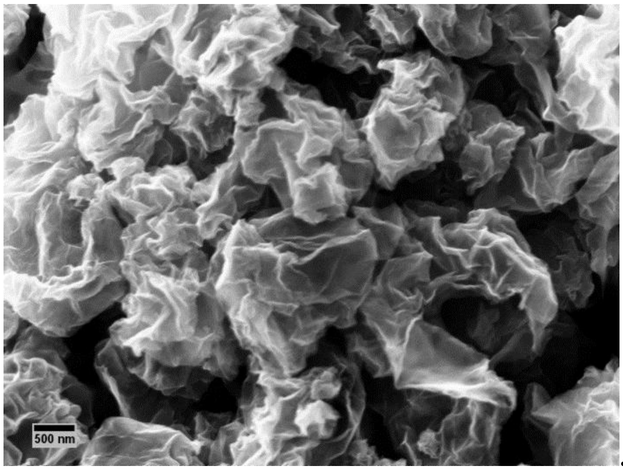

Figure S3. SEM micrographs of N-cGO(N_2+H_2) collected on Toray paper filter.

Table S2. D and G bands parameters obtained from the deconvolution of the Raman spectra of GO and of cGO, N-cGO, NS-cGO synthesized in inert and reductive atmosphere.

Sample	D band		G band		I_D/I_G
	Raman Shift (cm^{-1})	FWHM (cm^{-1})	Raman Shift (cm^{-1})	FWHM (cm^{-1})	
GO	1350	140	1580	65	1.04
cGO(N_2)	1355	142	1585	93	1.25
cGO(N_2+H_2)	1356	141	1584	83	1.19
N-cGO(N_2)	1344	120	1581	77	1.08
N-cGO(N_2+H_2)	1344	122	1580	68	1.08
NS-cGO(N_2) L-cysteine	1351	122	1580	65	1.13
NS-cGO(N_2+H_2) L-cysteine	1346	106	1582	65	1.15
NS-cGO(N_2) thiourea	1352	124	1585	68	1.09
NS-cGO(N_2+H_2) thiourea	1343	96	1582	62	1.08
Note: instrument resolution 5 cm^{-1}.					

References

[1] Marcano, D. C., Kosynkin, D. V., Berlin, J. M., Sinitskii, A., Sun, Z., Slesarev, A., Alemany, L. B., Lu, W., Tour, J. M. Improved Synthesis of Graphene Oxide. *ACS Nano* **2010**, *4*, 4806.
